# MALIGNANT HYPERTHERMIA IN A CHILD AFTER MAGNETIC RESONANCE IMAGING: A CASE REPORT

**DOI:** 10.1590/1984-0462/2020/38/2018267

**Published:** 2020-02-14

**Authors:** Carlos Gustavo de Almeida, José Colleti

**Affiliations:** a Hospital Assunção, São Bernardo do Campo, SP, Brazil.; b Hospital Santa Catarina, São Paulo, SP, Brazil.

**Keywords:** Malignant hyperthermia, Sevoflurane, Dantrolene, Hipertermia maligna, Sevoflurano, Dantroleno

## Abstract

**Objective:**

To report on a case of malignant hyperthermia in a child after a magnetic resonance imaging of the skull was performed using sevoflurane anesthesia.

**Case description:**

A 3-year-old boy admitted to the pediatric intensive care unit after presenting clinical and laboratory findings consistent with unspecified viral meningoencephalitis. While the patient was sedated, a magnetic resonance imaging of the skul was performed using propofol followed by the administration of sevoflurane through a laryngeal mask in order to continue anesthesia. Approximately three hours after the start of the procedure, the patient presented persistent tachycardia, tachypnea, generalized muscular stiffness and hyperthermia. With a diagnostic hypothesis of malignant hyperthermia, dantrolene was then administered, which immediately induced muscle stiffness, tachycardia, tachypnea and reduced body temperature.

**Comments:**

Malignant hyperthermia is a rare pharmacogenetic syndrome characterized by a severe hypermetabolic reaction after the administration of halogenated inhalational anesthetics or depolarizing muscle relaxants such as succinylcholine, or both. Although it is a potentially fatal disease, the rapid administration of continued doses dantrolene has drastically reduced the morbidity and mortality of the disease.

## RESUMO

**Objetivo:**

Relatar um caso de hipertermia maligna em criança após exame de ressonância magnética de crânio realizada sob efeito anestésico de sevoflurano.

**Descrição do caso:**

Menino de três anos de idade, admitido na Unidade de Terapia Intensiva Pediátrica (UTIP) após apresentar quadros clínico e laboratorial compatíveis com meningoencefalite viral não especificada. Foi realizado um exame de ressonância magnética de crânio com sedação utilizando, na indução anestésica, o propofol seguido pela administração de sevoflurano por meio de máscara laríngea para manutenção anestésica. Aproximadamente três horas após o início do procedimento, o paciente apresentou taquicardia, taquipneia, rigidez muscular generalizada e hipertermia persistentes. Com hipótese diagnóstica de hipertermia maligna, foi então administrado dantrolene, que fez ceder de forma imediata a rigidez muscular, a taquicardia, a taquipneia e reduziu a temperatura corporal.

**Comentários:**

A hipertermia maligna é uma síndrome farmacogenética rara, que se caracteriza por reação hipermetabólica grave após administração de anestésicos inalatórios halogenados e/ou relaxantes musculares despolarizantes, como a succinilcolina. Apesar de ser uma doença potencialmente fatal, a rápida administração de dantrolene, junto às doses de manutenção, tem reduzido drasticamente a morbimortalidade da doença.

## INTRODUCTION

Malignant hyperthermia (MH) is a potentially fatal pharmacogenetic syndrome triggered by the administration of halogenated anesthetics (ie: halothane, isoflurane, sevoflurane) and neuromuscular blockers such as succinylcholine.^1^ ^,^ ^2^ Following exposure to such drugs, genetically predisposed patients develop contractures and their skeletal muscles become rigid. This results from the sarcoplasmic reticulum-releasing calcium ions. These contractures are so intense that they promote increased oxygen consumption, excess CO_2_ production, hyperthermia from the production of excess heat (an increase of 1 to 2°C every five minutes) and rhabdomyolysis. Moreover, it has associated symptoms such as tachypnea and tachycardia.^1^ ^,^ ^2^ ^,^ ^3^

Treatment includes immediate discontinuation of the triggering agent, 100% oxygen delivery, hyperventilation (when on mechanical ventilation), temperature control, and prompt administration of dantrolene intravenously.^1^ ^,^ ^2^ ^,^ ^3^ Prior to the advent and use of dantrolene, mortality associated with MH was approximately 80%. However, now after it has been used, mortality has been reduced to approximately 5%.^1^

We report the case of a three-year-old patient with a clinical presentation of MH who responded adequately to dantrolene, but the symptoms increased because he did not continue taking the drug in the proper therapeutic sequence. After taking dantrolene again, the patient’s clinical condition was controlled.

## CASE REPORT

A three-year-old male was admitted to the Pediatric Intensive Care Unit (PICU) because of dizziness, vomiting, dysarthria and seizures. Meningoencephalitis was suspected, and thus a cerebrospinal fluid exam was performed, which showed increased cellularity with 70% neutrophils cells, 30% lymphomononuclear cells, glucose 60 mg/dL, proteinorrhage 55 mg/dL and a positive Pandy’s test. The bacterioscopy, latex reaction, culture tests and Herpes virus serology were all negative. He was treated with the diagnostic of an unspecified viral meningoencephalitis with acyclovir at a dose of 30 mg/kg/day for 14 days, due to clinical improvement after starting the medication. After the infection was stabilized, the neuro-pediatric team requested a magnetic resonance imaging (MRI) of the child’s cranium because he was already in the infirmary ward. The MRI was performed under Propofol-induced anesthetic effect and maintained with sevoflurane through a laryngeal mask. No opioids, muscle relaxants or benzodiazepines were administered. The child tolerated the procedure well, and was transported back to the infirmary ward after recovering from the anesthesia. Approximately three hours after the procedure, the patient presented tachycardia, tachypnea, generalized muscle stiffness (especially in the masseter), and hyperthermia, in that order.

**Graph 1:**
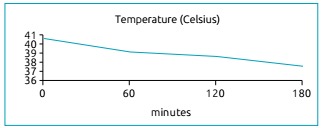
Thermal curve after the administration of dantrolene

Initially, seizures were considered to be the cause due to his underlying pathological condition (meningoencephalitis). However, after receiving paranasal catheter oxygen therapy and two doses of diazepam at 0.1 mg/kg/dose, his symptoms persisted. The child was again referred to the PICU. Both the pediatric anesthesia and neurology teams, made a diagnosis of MH, which was considered late, received 48 points in the MH Risk Rating Scale (Tables 1 and 2), and was classified as “very likely”. Following intravenous administration of dantrolene at a dose of 2.5 mg/kg, the child responded effectively and the symptoms disappeared almost immediately. His hyperthermia subsided (Graph 1), as well as muscle contractions and other symptoms. Approximately 15 minutes after the dantrolene was administered, his symptoms already under control, and laboratory tests were performed (Table 3). Among the laboratory findings, the changes related to MH found in the patient were hypoxemia (partial arterial oxygen pressure - PaO_2_ 63 mmHg), hyperlactatemia (26.9 mg/dL) and an elevation of muscle enzymes (creatine kinase - CK 1592 U/L).

**Table 1:** Clinical classification scale for malignant hyperthermia.

Pathophysiological Process	Indicators	Score
1) Muscle stiffness	a) Generalized b) Masseter after succinylcholine	a) 15 b) 15
2) Muscle destruction	a) CPK> 20,000 IU with succinylcholine b) CPK> 10,000 IU without succinylcholine c) Dark urine d) Myoglobinuria> 60 µg / L e) Myoglobinemia> 170 µg / L f) Potassemia> 6 mEq / L	a) 15 b) 15 c) 10 d) 5 e) 5 f) 5
3) Respiratory acidosis	a) PET_CO2_> 55 mmHg at proper MPV b) PET_CO2_> 60 mmHg in spontaneous ventilation c) Pa_CO2_> 60 mmHg at proper MPV d) Pa_CO2_> 65 mmHg in spontaneous ventilation e) Inappropriate hypercapnia f) Inappropriate tachypnea	a) 15 b) 15 c) 15 d) 15 e) 15 f) 15
4) Hyperthermia	a) Rapid and inappropriate rise in temperature b) Temperature> 38.8 ° C (not appropriate)	a) 15 b) 10
5) Heart rhythm	a) Inappropriate sinus tachycardia b) Tachycardia or ventricular fibrillation	a) 3 b) 3
6) Administration of dantrolene	a) Rapid reversal of symptoms	a) 5
7) Acidemia	a) arterial BE (-8 mEq/L) b) arterial pH <7.25	a) 10 b) 10
Score	Risk	Rating
0	Risk 1	Almost impossible
3 to 9	Risk 2	Unlikely
10 to 19	Risk 3	Less than likely
20 to 34	Risk 4	More than likely
35 to 49	Risk 5	Fairly likely
50 or more	Risk 6	Almost certain

Source: Raut et al. ^2^

CPK: Creatine Phosphokinase; PET_CO2_: end-tidal carbon dioxide partial pressure; MPV: mechanical pulmonary ventilation; Pa_CO2_: arterial carbon dioxide pressure; °C: Celsius; BE: base excess.

**Table 2:** Reported patient clinical indicators based on the rating scale.

Pathophysiological Process	Score
1. Generalized muscle stiffness	15
2. Inappropriate tachypnea	10
3. Inappropriate hyperthermia	15
4. Inappropriate tachycardia	3
5. Fast reversal after dantrolene	5
*Total score*	*48*
Probability	Risk for malignant hyperthermia: 5 or Fairly likely

**Table 3:** Laboratory tests of the patient on presenting malignant hyperthermia.

Laboratory Variable	Patient Outcome	Normality Limits
Arterial pH	7.44	7.35 to 7.45
Pa_O2_ arterial (mmHg)	63	80 to 100
Pa_arterial CO2_ (mmHg)	37	35 to 45
HCO_3_ arterial (mmol/L)	24	22 to 26
Arterial BE	0	-3.0 to +3.0
Plasma lactate (mg/dL)	26.9	5,7 to 22,0
Plasma CPK (U/L)	1,592	30 to 150
Plasma CPK-MB (ng/mL)	35	Less than 5
Myoglobinuria	negative	negative
Plasma K (mEq/L)	3.9	3.5 to 4.5

Pa_O2_: partial pressure of arterial oxygen; Pa_CO2_: partial pressure of arterial carbon dioxide; HCO_3_: sodium bicarbonate; BE: base excess; CPK: Creatine Phosphokinase; CPK-MB: MB Fraction of Creatine Phosphokinase; K: potassium.

Even though the patient evolved well, two days later his symptoms returned, probably because he did not maintain the doses. Dantrolene had to be administered again and maintained at a dose of 0.5 mg/kg every 12 hours. After starting doses that were maintained, the patient’s clinical condition was stabilized again. Dantrolene was administered continuously for 48 hours without further seizures.

After the diagnosis became clear and effective treatment was given, the patient’s family members were advised about the need for follow-up at the Center for Study, Diagnosis, and Investigation of Malignant Hyperthermia of the Universidade de São Paulo (CEDHIMA/Unifesp), which also provides guidance via a telephone hotline to assist healthcare professionals in diagnosing and treating MH.

## DISCUSSION

We are reporting on the case of a pediatric patient who had a clinical presentation of MH in the pediatric ward after being given sevoflurane anesthesia in order to perform a magnetic resonance imaging diagnostic procedure. He responded well to the administration of dantrolene. However, symptoms recurred after the drug was not administered in the post-crisis period, which were maintenance doses. After restarting dantrolene therapy, the symptoms that had relapsed were controlled.

The incidence of MH episodes during anesthesia is between 1:10,000 and 1:250,000 and, specifically in pediatric patients, 1:15,000.^1^ ^,^ ^4^ Although MH may be triggered after a first exposure to anesthesia with known agents, on average, these patients require three exposures to such anesthetics before it is triggered. Male patients are most affected at a 2:1 ratio. Additionally, pediatric patients under 15 years old make up 52% of reported cases.^1^

MH is known to be an autosomal dominant genetic disease and, in certain situations, may be associated with certain congenital myopathies.^1^ ^,^ ^2^ ^,^ ^3^ In up to 50% of predisposed patients, the causative mutation of this syndrome occurs in the gene that encodes the ryanodine receptor (RYR1), which is responsible for releasing calcium from the sarcoplasmic reticulum of the muscle fiber. However, it is known that this syndrome has a very varied genotype and is also associated with a mutation in the CACNA1S gene, which is the gene responsible for the skeletal muscle calcium channel.^1^ ^,^ ^3^ ^,^ ^5^ A contracture test in vitro in a recent skeletal muscle biopsy determines whether a patient is susceptible. The contractures are measured in the presence of halothane and caffeine, and a “Gold standard” evaluation for the diagnosis of MH is considered.^1^ ^,^ ^6^ ^,^ ^7^ Molecular testing can also be used as a diagnostic measure, especially in Europe and the United States, but its high cost and technical difficulty have limited its use.^5^ ^,^ ^8^ However, no molecular test can replace the muscle contracture test in vitro, due to the disease’s heterogeneity.^6^ ^,^ ^7^

Fundamental clinical features of MH include sustained muscle stiffness, stiffness in the masseter muscle, accompanied by hyperthermia, which may exceed 41°C. This begins after exposure to some of the anesthetic drugs already mentioned.^1^ ^,^ ^2^ ^,^ ^4^ ^,^ ^9^ Tachycardia and tachypnea also occur in conjunction with these manifestations. In the absence of an appropriate and prompt medical intervention, the following may occur: rhabdomyolysis - with a risk of myoglobinuria - kidney damage, hyperkalemia, cardiac arrhythmias, and death.^1^ ^,^ ^3^ ^,^ ^4^ ^,^ ^10^

The clinical manifestations that were present in the reported patient and which are part of the diagnostic criteria for MH are: generalized and masseter muscle stiffness, tachycardia, tachypnea, and hyperthermia (Tables 1 and 2). The laboratory tests of the patient (Table 3) did not show the classic metabolic acidosis or hypercapnia usually present in MH. However, there was a considerable increase in muscle enzymes, hyperlactatemia and arterial oxygen desaturation. There were no laboratory abnormalities related to renal function, such as hyperkalemia or the presence of myoglobinuria. Despite having tachycardia with an elevated MB fraction of creatine phosphokinase (CPK-MB), no arrhythmias or cardiac dysfunctions were reported in the electrocardiogram performed. All clinical and laboratory findings found in the patient are part of the diagnostic criteria approved by *Projeto Diatrizes*, a joint initiative of the Brazilian Medical Association (*Associação Médica Brasileira* - AMB) and the Federal Council of Medicine (*Conselho Federal de Medicina* - CFM), which aims to reconcile information from the medical field, in order to standardize conduct that help doctors reason and make decisions. ^9^ ^,^ ^11^

Most of the time, the manifestations of malignant hyperthermia occur suddenly and early on. The syndrome can be fatal when not treated properly, leading the patient to have intense muscle catabolism and its serious consequences, especially at the respiratory and cardiovascular level.^3^ The late form of the disease, as happened with this patient, is very rare - less than 2% of cases - and has been described as occurring within 4.5 hours after anesthetic trigger interruption, presenting with more attenuated clinical manifestations, but a similar severity to the early form, due to complications. Despite its severity, delayed MH rarely reaches a fulminant crisis.^2^ ^,^ ^3^ ^,^ ^9^

It is of utmost importance that patients with MH be monitored in an intensive care setting for a minimum of 48 to 72 hours, as symptoms may recur in up to 25% of cases. Continuing dantrolene treatment has been proposed. This drug inhibits calcium channel receptors in skeletal muscles, and restores balance in the sarcoplasmic reticulum for up to four to six hours after administration. Therefore, intermittent doses of up to 1 mg/kg should be given at intervals of up to six hours within 48 hours of the initial event or continuously infused at a dose of 0.25 mg/kg/hour for 24 hours.^1^ ^,^ ^2^ ^,^ ^8^ ^,^ ^10^ The patient received the dantrolene loading dose after a clear diagnosis, and his symptoms improved rapidly. However, because maintenance doses were not continued, symptoms recurred and dantrolene was prescribed again for 48 hours in intermittent doses, thus controlling the symptoms.

Auxiliary treatments should be instituted immediately after the administration of dantrolene, such as rapid temperature cooling with infusion of 15 mL/kg cold saline, 100% oxygen delivery with mild hyperventilation (when on mechanical ventilation), end-tidal carbon dioxide concentration (ET_CO2_) through capnograph and acidosis control.^3^ ^,^ ^4^ ^,^ ^8^ The patient should be referred to the PICU for monitoring and treatment of possible complications such as cardiac arrhythmias due to hyperkalemia and acute renal injury due to myoglobinuria.^3^ ^,^ ^10^

It is worth noting that the importance of different diagnoses, considering that MH can be confused with febrile seizures, especially in children in whom such seizures are very common until the age of five. Other diseases with hypercatabolism should be ruled out, such as thyrotoxicosis, pheochromocytoma, sepsis, pyrogenic shock and some drugs, especially those that cause extrapyramidal symptoms.^1^ ^,^ ^4^ ^,^ ^9^ There are also reports that susceptibility to MH increases in patients with certain congenital myopathies due to their association with mutation in the RYR1 gene. Medical professionals’ prior knowledge of this information is essential in order to optimize the clinical care of these patients when they need surgery, as proposed by Bamaga et al.^1^ ^,^ ^3^ ^,^ ^11^

In order to assist health professionals and victims’ families, a service for MH was created in Brazil in 1991 called Hotline (+ 55-11-55759873). It is located in the city of São Paulo, specifically at the Hospital São Paulo, which is part of Unifesp’s Paulista School of Medicine (*Escola Paulista de Medicina* - EPM). It is a 24-hour MH hotline service, coordinated by a group of two MH research supervisors - a neurologist and an anesthesiologist - as well as other medical, biomedical, and nursing staff. All of them have received training that consisted of an intensive MH course, with theoretical and practical information for the diagnosis, treatment and follow-up of patients with MH through Unifesp’s CEDHIMA.^12^ ^,^ ^13^

It can be concluded that MH is a rare and serious disease for susceptible individuals undergoing anesthesia using volatile agents. Early recognition of signs and symptoms for proper treatment is of paramount importance, as delayed diagnosis and treatment increases morbidity and mortality from the disease. In addition, the immediate use and maintenance of dantrolene for a period of no less than 48 hours is essential for the survival of these patients.
